# Human Papillomavirus (HPV) Vaccine Utilization and Its Determinants Among Adolescents in the United Arab Emirates (UAE): A Review

**DOI:** 10.7759/cureus.93179

**Published:** 2025-09-25

**Authors:** Sarah I Zahid, Bushra Alnuaimi, Kifaya Tamimi, Nadia I Zahid, Latifa Al Saad, Mazaffar I Zahid, Jayakumary Muttappallymyalil, Jayadevan Sreedharan

**Affiliations:** 1 Internal Medicine, Gulf Medical University, College of Medicine, Ajman, ARE; 2 Internal Medicine, University of Sharjah, College of Medicine, Sharjah, ARE; 3 Family Medicine, Nassau University Medical Center, East Meadow, USA; 4 Epidemiology and Public Health, Mohammed Bin Rashid University of Medicine and Health Sciences, Dubai, ARE; 5 Community Medicine, Thumbay Institute of Population Health, Gulf Medical University, Ajman, ARE

**Keywords:** hpv, hpv vaccine, human papillomavirus, human papillomavirus vaccine, immunization, united arab emirates, vaccine uptake

## Abstract

Human papillomavirus (HPV) can cause a range of diseases, the most common being cervical cancer. Although vaccines have existed for decades and are highly effective at preventing the illness, vaccination coverage is low, specifically among adolescents. There has been some success in vaccination campaigns in the UAE among girls, but very low male adolescent vaccination rates. It is essential to identify vaccination patterns as well as predictors of uptake to improve public health outcomes.

This narrative review included relevant studies published between 2005 and 2025 in the PubMed, Scopus, Google Scholar, ScienceDirect, and Embase databases. The MeSH terms used were "Human Papillomavirus vaccine," "HPV vaccine," "Human Papillomavirus," "HPV," "vaccine uptake," "immunization," and "United Arab Emirates."

Following the introduction of HPV vaccination in adolescent girls in 2008, the UAE saw a dramatic expansion of coverage, with some areas exceeding 90%. However, recent trends show the number of adolescents initiating vaccination has declined while completion is rising. Male data are limited but demonstrate very low coverage. Determinants of the uptake of vaccines include awareness levels, religious and cultural attitudes, fear of side effects, and concerns about costs and availability of the vaccine. Doctor referral was an extremely powerful motivator for vaccination. The UAE is behind its immediate regional neighbors in the uptake of vaccines, but leads when compared to international standards.

While the UAE has shown tremendous improvement in HPV vaccination, there remains some room for growth, especially in male adolescents. Expanding education, drifting from cultural resistance to vaccination, and increasing vaccine availability are measures that are crucial to optimizing vaccine distribution and reducing HPV-related illnesses in the years ahead.

## Introduction and background

Human papillomavirus and its public health relevance

HPV is one of the most prevalent sexually transmitted infections (STIs) globally, affecting both men and women. Over 200 genotypes of HPV have been identified, with at least 17 considered high-risk due to their strong association with cancer development [1]. Among these, HPV types 16 and 18 are responsible for approximately 77% of cervical cancer cases worldwide [2]. Persistent infection with these high-risk types can lead to a range of malignancies, including cervical, anal, vulvar, vaginal, penile, and oropharyngeal cancers [3,4]. Cervical cancer remains a major global health concern. In 2020, it ranked as the fourth most common cancer among women, with over 600,000 new cases and 340,000 deaths annually, disproportionately affecting low- and middle-income countries [5]. The burden of HPV-related cancers is not limited to women. Approximately 90% of anal cancers, 63-80% of oropharyngeal cancers, and up to 50% of penile cancers are also attributed to HPV, highlighting its public health significance across both sexes [6,7].

HPV infection is a public health concern in the United Arab Emirates (UAE), where cervical cancer is still the most common cancer among females, with an annual incidence of 7.4 per 100,000 [8-11]. HPV and its connection with cervical cancer are not widely known among women in the UAE since only 29% have heard of the infection, and fewer are aware of its association with cervical cancer or its vaccine [12,13]. Molecular epidemiology studies have reported a high prevalence of both low- and high-risk HPV genotypes, with recent data showing that over 60% of women in certain cohorts test positive for HPV, including oncogenes such as HPV 16, HPV 45, and HPV 51 [14,15]. Co-infection with multiple genotypes is common, and a high percentage of women with normal cytology are positive for high-risk HPV, emphasizing the potential for developing cervical neoplasia [14,15].

Regional reports confirm that high-risk HPV in the UAE shares similarities with fellow members of the Gulf Cooperation Council [16]. The disease burden of HPV is accompanied by poor public awareness and suboptimal utilization of cervical cancer screening programs that are not fully covered on a national level [9]. Even though the UAE's cervical cancer prevalence is lower than the global average, the absolute figure of cancers due to HPV will rise because of population growth and changes in the risk factors. Such risk factors include shifts in sexual habits and the gaps in preventative health education [10,11]. This highlights the need for epidemiological surveillance, increased screening protocols, and targeted public health interventions to address the prevalence of HPV and its consequences in the UAE [12-16].

In response to the global HPV burden, the World Health Organization (WHO) has recognized HPV vaccination as a critical intervention in cancer prevention and launched a global strategy to eliminate cervical cancer as a public health problem by 2030 [17]. The introduction of prophylactic vaccines such as the bivalent (Cervarix), quadrivalent, and nonavalent (Gardasil 9) has significantly changed the landscape of HPV prevention. These vaccines have demonstrated high efficacy in preventing infection with oncogenic HPV types, especially when administered before the onset of sexual activity [18]. Evidence from countries with robust vaccination programs, such as Australia, the United Kingdom, and the United States, shows a marked decline in HPV infections, genital warts, and precancerous cervical lesions over time, underscoring the vaccine’s impact on population health [19,20]. Additionally, widespread HPV vaccination indirectly protects unvaccinated individuals through herd immunity, further amplifying its public health benefits [21].

Despite its proven safety and effectiveness, global HPV vaccine coverage remains variable. Challenges such as limited awareness, vaccine hesitancy, cultural beliefs, and healthcare access disparities continue to affect uptake, particularly in regions like the Middle East and North Africa (MENA), including the UAE [22,23]. Therefore, increasing HPV vaccine coverage through national programs, education campaigns, and healthcare system support is essential to reduce the burden of HPV-related diseases and improve long-term health outcomes.

HPV-related diseases

HPV causes a variety of diseases that can be largely prevented with vaccination. The virus is a non-enveloped, double-stranded, circular DNA virus with more than 200 subtypes; the four most discussed are HPV 6, HPV 11, HPV 16, and HPV 18. Subtypes 6 and 11 are low risk and can manifest as low-grade precancerous lesions, condyloma acuminatum or anogenital warts, and recurrent respiratory papillomatosis, commonly due to vertical transmission during childbirth. Subtypes 16 and 18 are high risk, which manifest as high-grade intraepithelial lesions that can lead to malignancies [24-27]. Such examples include head and neck cancer, anal squamous cell carcinoma, penile squamous cell carcinoma, and cervical squamous cell carcinoma [28-31]. Though HPV is transmitted through sexual contact, it should be noted that other risk factors are present for the presentation of the virus. Risk factors include smoking, folate deficiency, more than three pregnancies, immunosuppression, UV light exposure, being sexually active at an early age, history of sexually transmitted diseases, and the number of sexual partners [24,32]. Due to the screening programs involving Pap smears, fluid-based cytology, and the Bethesda System reporting system, the cervical cancer incidence and mortality have declined. Nonetheless, a vaccination regimen against HPV is presented as a solid resolution in decreasing rates of HPV-related diseases [33].

Global overview of HPV vaccination programs

HPV is an indispensable intervention for the purpose of preventing cervical cancer and other related diseases, such as genital warts or other cancers affecting the oropharynx. In fact, around 147 countries introduced this vaccine as of December 2024 [34]. According to the World Health Organization’s global strategy to accelerate the elimination of cervical cancer, one of their many goals is to reach 90% HPV vaccination coverage in girls by the age of 15 [35]. Moreover, a routine vaccination is recommended by the WHO among girls aged 9-14 due to their efficient immune response before exposure to sexual activity [36]. An increasing number of high-income nations have augmented their HPV vaccination program to be one that is gender-neutral in order to protect both girls and boys against any HPV-related diseases [37]. Additionally, 57 supported, lower-income countries showcased an increase in HPV vaccination coverage of 16% from 2019 to 2023 [38]. Though there have been improvements in this vaccine’s coverage, obstacles and barriers remain, such as misconceptions and knowledge gaps regarding HPV and its vaccine [39] as well as healthcare workforce barriers [40], providing the opportunity to mitigate them and encourage significant HPV vaccination utilization. This review aimed to assess the utilization of the HPV vaccine and the factors influencing its uptake among adolescents in the UAE and identify important key determinants that have an influence on the uptake of the HPV vaccine among this population, providing an insight for health promotion and policies.

Rationale and objective

HPV infection is a well-established cause of cervical cancer and other HPV-related diseases. Vaccination during adolescence, prior to the onset of sexual activity, is globally recognized as the most effective strategy for preventing HPV infections. The UAE introduced the HPV vaccine as part of its national immunization program, primarily targeting school-aged girls. Despite this, there is limited evidence regarding the extent of vaccine uptake and the factors influencing acceptance and utilization, particularly among adolescents.

## Review

Methodology

**Figure 1:**
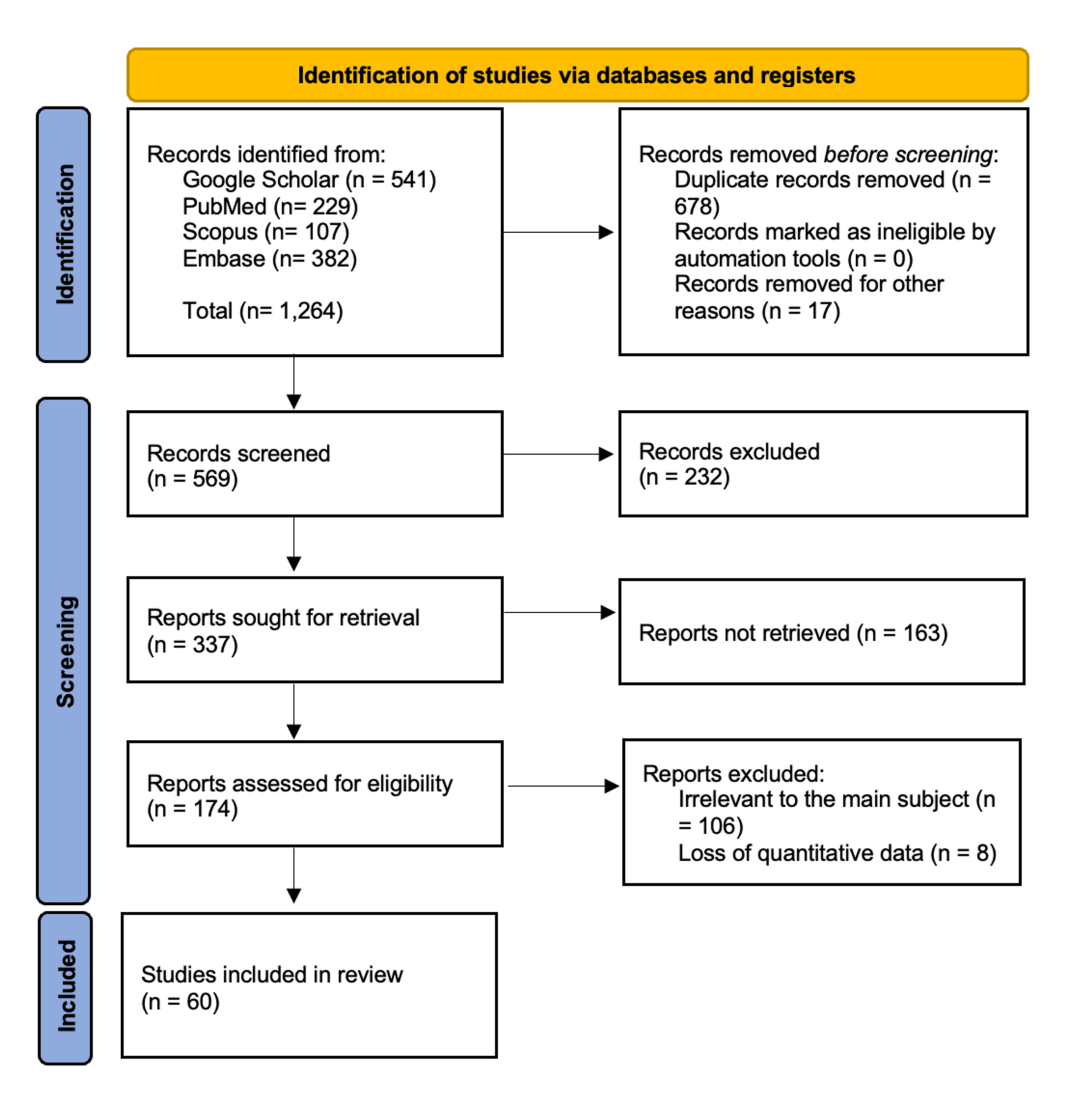
PRISMA of Study Selection

This review conforms to the “Preferred Reporting Items for Systematic Reviews and Meta-Analyses” (PRISMA) statement (Figure 1). The summary table of the included studies is provided (Table 1).

**Table 1:** Summary Table of the Included Studies

Author(s)	Year	Population	Design	Outcomes	Key Findings
Schiffman M, Castle PE	2005	Global	Commentary	Cervical cancer prevention potential	HPV vaccination & screening can eliminate cervical cancer
Ahmed HG et al.	2017	Yemeni women with cervical cancer	Cross-sectional	Prevalence of HPV16,18	HPV 16 (37%) and HPV 18 (16%) are the most predominant HPV subtypes; underscoring the need for vaccination in limited resource settings
Forman D et al.	2012	Global	Systematic review	Burden of HPV related disease	HPV causes around 5.2% of cancers globally and cervical cancer is the most common malignancy in HPV
Gillison ML et al.	2008	General population	Review	Noncervical HPV cancers	HPV can cause oropharyngeal, anal, penile, vulvar cancers and may be prevented by vaccines.
World Health Organization	2024	Global	Fact sheet	Burden of Cervical cancer	There are around 660k new cases of cervical cancer in 2022, 350k deaths in 2022. Most cases of incidence and mortality occur in low- and middle-income countries.
CDC	2023-2025	US population	Public Health report	HPV related cancers	HPV causes around 10,800 cancers per year in the US and most can be prevented by vaccines.
Giuliano AR et al.	2008	Men globally	Review	HPV in men and noncervical cancers	There is a high prevalence of HPV in men, related to oropharyngeal, anal, and penile cancers
Elbarazi I et al.	2016	UAE	Content Analysis	HPV vaccine's media portrayal	HPV vaccine was framed negatively by Arabic media, while English media represented it to be more neutral/positive.
Vaccarella S et al.	2013	MENA region	Review	Burden of HPV in MENA region	Burden of HPV is underestimated with low screening rates. HPV 16,18 is dominant.
Ortashi O et al.	2013	UAE women	Cross-sectional	Awareness and knowledge	Awareness of HPV is 60%, 45% can link it to cervical cancer, and there’s also low understanding of the transmission/vaccination.
Zhang J et al.	2025	Global	Modelling Study	Burden of HPV cancer by site	Rising Anogenital and oropharyngeal cancers with variation of HPV type among regions.
Alsous MM et al.	2021	Arab women	Cross sectional	Knowledge and awareness of HPV and its vaccination	knowledge of HPV link to cancer was 43.8% and only 20.3% knew vaccine could prevent cervical cancer
Odeh HI et al.	2023	UAE women	Pilot study	Prevalence of HPV genotype	Most common HPV genotypes: 6, 45, 16, 11, 67, 62/81. Calls for new vaccines to reduce the burden of cervical infection
Krishnan K, Thomas A	2016	UAE women	Retrospective cohort	HPV genotypes, cytology, and histology	Correlating cervical cytology with HPV genotyping and histopathology is a vital control measure.
Ali MAM et al.	2019	GCC women	Systematic review	HPV prevalence and types	positive correlation between HPV+ and abnormal cytology
Shoja Z et al.	2019	WHO EMRO region	Meta analysis	HPV type distribution	74.5% of cervical neoplasia was prevented in EMRO due to HPV vaccination and screening.
WHO	2020	Global	Policy document	Elimination of Cervical cancer strategy	Global strategy: 90-70-90, 90% of girls vaccinated, 70% of women screened, 90% with cervical disease receive treatment
Garland SM et al.	2007	Women 16-26 years	RCT	Efficacy of quadrivalent HPV vaccine	HPV quadrivalent vaccine significantly reduced HPV related anogenital disease incidence.
Hall MT et al.	2019	Australia	mathematical modeling	Cervical cancer elimination timeline	Australia projected to reduce incidence of cervical cancer to less than 4 per 100,000 in women by 2028 and less than 1 by 2066
Drolet M et al.	2019	Global (post vaccination)	Systematic review & meta-analysis	Herd immunity, population impact	HPV vaccination programs have greatly lowered the prevalence of high-risk HPV subtypes, warts, and cervical intraepithelial neoplasia
Brisson M et al.	2020	US population	Model-based analysis	Impact of vaccination strategies	Significant drop in genital warts and precancerous cervical lesions 5-9 years after HPV vaccination introduction
Sallam M et al.	2021	Arab countries	Systematic review	Knowledge and acceptance of HPV	Low HPV vaccine acceptance in MENA region due to limited awareness, cost, beliefs, and safety concerns.
Ortashi O et al.	2020	UAE male university students	Cross sectional	Acceptance of HPV vaccine	Low acceptability and knowledge of HPV infection and its vaccination.
Luria L, Cardoza-Favarato G	2023	General population	Review	HPV biology, transmission	HPV is a prevalent DNA virus and can cause benign warts and cervical cancer (subtypes 16,28), via oncoproteins E6 and E7.
Pennycook KB, McCready TA	2023	General population	Review	Condyloma acuminata	Condyloma acuminata caused mainly by HPV 6, 11, can lead to anogenital warts and can be prevented by vaccination
Venkatesan NN et al.	2012	Patients with RRP	Clinical review	Recurrent respiratory papillomatosis	RRP caused primarily by HPV 6, 11 with airway symptoms, managed by surgical removal of lesions and adjuvant therapy.
Wichmann G	2017	Head and neck cancer patients	Review	HPV subtypes in HNSCC	High risk HPV subtypes produce E6 and E7 proteins vary in their neoplastic potential resulting in differences clinically in head and neck cancer
Mody MD et al.	2021	Head and neck cancer patients	Review	Epidemiology and management	Head and neck cancer is driven by smoking, alcohol, HPV, and necessitates multidisciplinary treatment.
Fowler JR et al.	2023	General population	Review	Cervical cancer diagnosis and treatment	Cervical cancer is mostly caused by HPV 16,18 and is greatly prevented by vaccine as well as screening
Iorga L et al.	2020	Penile cancer patients	Review	HPV and penile cancer	Penile carcinoma can be highly treatable earlier in its course and is mainly triggered by HPV 16,18
Iorga L et al.	2020	Patients with penile carcinoma	Review	Association of HPV with penile carcinoma	HPV-16 is the most common subtype; vaccination may help reduce incidence
Leon ME et al.	2015	Anal SCC case	Case Report	Papillary features of HPV-related anal SCC	HPV testing is important for proper diagnosis
Del Pino M et al.	2024	General population	Targeted Literature Summary	HPV infection and disease risk factors	Sexual behavior and immunosuppression are key risks
Williamson AL	2023	Global	Review	HPV vaccine advancements	Highlights newer nonavalent vaccines and coverage
VIEW-Hub	2025	Global	Data Report	HPV vaccine rollout and coverage	Provides country-level HPV vaccine implementation data
WHO	2024	Global	Fact Sheet	HPV and related cancers	HPV causes cervical, anal, penile cancers; vaccine effective
WHO	2022	Global	Position Paper	HPV vaccine recommendations	Provides official guidance on schedules and safety
Bruni L et al.	2022	Global	Data Analysis	HPV vaccine uptake (2010–2019)	Varied global uptake; corrections applied
GAVI Alliance	2025	LMICs	Web Resource	Support for HPV vaccine programs	Outlines vaccine funding and implementation mechanisms
Moreno VA et al.	2025	Central American immigrants in US	Cross-sectional	HPV knowledge and misconceptions	Major knowledge gaps; need for culturally sensitive education
Fousseni S et al.	2025	Sub-Saharan African countries	Systematic Review	Challenges in vaccine introduction	Barriers include funding gaps, poor infrastructure, and low awareness
Al-Nuaimi NS et al.	2011	Traditional UAE city population	Cross-sectional	HPV vaccine uptake	Low uptake due to lack of awareness and cultural factors
Al Shdefat S et al.	2022	Emirati men	Survey Study	HPV vaccine acceptability	Awareness low; willingness increases with doctor recommendation
HPV Centre	2023	UAE	Country Report	HPV stats and screening coverage	Provides UAE-specific data for policy and planning
WHO	2025	UAE and USA	Web Dashboard	HPV vaccine coverage by dose/gender	Real-time comparative statistics for monitoring programs
Mahmoud I et al.	2024	Young adults in GCC	Cross-sectional	HPV awareness and Pap test uptake	Low awareness, especially among males; highlights need for education
Alshahrani NZ et al.	2024	Parents in GCC	Systematic Review	Parental attitudes toward HPV vaccine	Cultural and religious concerns are common barriers
WAM	2025	UAE	News Report	WHO recognition of UAE's public health policies	UAE honoured for effective HPV-related public health campaigns
Senior S (Gulf News)	2024	School boys in UAE	News Article	New MoHAP school vaccine policy	Policy includes boys in HPV vaccination; aims for gender equity
Dept. of Health UAE	2025	UAE	Govt Report	Late-stage cervical cancer burden	Calls for earlier screening and improved HPV vaccine uptake
Burjeel Hospital	2022	UAE women	News Article	Cervical cancer trend	Dramatic decline due to proactive screening and vaccination policies
Khaleej Times	2025	UAE parents	News Report	Willingness to vaccinate children	Growing parental acceptance linked to public awareness and trust
Wang S et al.	2020	Male university students	Cross-sectional	HPV knowledge	Low awareness; emphasizes need for targeted education for men
Garcia MR et al.	2024	General	Review Chapter	Overview of HPV as STI	HPV causes cancer and genital warts; vaccine crucial for prevention
Kurt A et al.	2024	Parents (Turkey)	Cross-sectional	Parental HPV beliefs	Religious and cultural beliefs influence vaccine acceptance
AlMesbah N et al.	2024	GCC countries	Scoping Review	HPV prevalence and genotype distribution	HPV-16 and -18 are most common in GCC; supports vaccine need
Kisa S, Kisa A	2024	Islamic countries	Scoping Review	Religious attitudes toward HPV vaccine	Acceptance linked to religious guidance and community awareness
Barqawi HJ et al.	2024	UAE parents	Cross-sectional	Vaccine literacy and hesitancy	Low literacy; recommends physician-driven education
Tobaiqy M, MacLure K	2024	Global	Systematic Review	HPV vaccination barriers and strategies	Challenges include cost, misinformation; strategies include school mandates and outreach
Gebreal A et al.	2025	Eastern Mediterranean Region	Systematic Review & Meta-analysis	Parental willingness to vaccinate children	Education, physician advice, and affordability improve uptake
Ayash C et al.	2024	Healthcare providers serving Arab American populations	Qualitative Study	HPV vaccination barriers, knowledge, and provider practices	HPV vaccination barriers, knowledge, and provider practices

Search Strategy

The authors searched the electronic databases PubMed, Scopus, Google Scholar, ScienceDirect, and Embase, published between 2005 and 2025, to get articles included in this narrative review, covering all available years of publication up to that date. Additionally, reference lists of the included articles were manually screened for relevant studies. The search strategy included the Medical Subject Headings (MeSH) terms "Human Papillomavirus vaccine," "HPV vaccine," "Human Papillomavirus," "HPV," "vaccine uptake," "immunization," and "United Arab Emirates." All studies on HPV vaccine utilization and its determinants among adolescents in the UAE were included. After getting the articles, the authors screened the relevant papers to be included in this narrative review.

Study Selection and Data Extraction

Following the search, article citations were imported into the reference manager, EndNote, and duplicates were removed. The researchers were divided into two groups with comparable experience and expertise to minimize the chance of missing relevant sources. Citations were independently evaluated by both groups by title and abstracts in the first stage of study selection, followed by a full-text review of the selected articles. Reasons for the exclusion of full-text studies were recorded. Any reference conflicts between the groups were resolved by consensus regarding the eligibility to include in the review.

In this narrative review, studies were selected based on specific inclusion and exclusion criteria to ensure methodological rigor and relevance. Articles published between 2005 and 2025 that examined human papillomavirus (HPV) vaccine utilization and its determinants among adolescents in the United Arab Emirates were included. Eligible study designs comprised cross-sectional studies, reviews, case reports, meta-analyses, health reports, and experimental studies. Only full-text, peer-reviewed publications indexed in major databases were considered. Excluded were interventional studies unrelated to HPV vaccination, studies lacking or reporting inappropriate designs, and those irrelevant to HPV vaccination or missing quantitative data.

Results

National Statistics and Trends Over time

It is important to recognize the national statistics and trends over time in the HPV vaccine uptake among adolescents in the UAE. HPV vaccination was launched and promoted first in Abu Dhabi in 2008, targeting female students aged 15-17 years entering grade 11 or grade 12. This was promoted through a free school-based campaign, with the initial uptake being reported to be approximately 53% within Abu Dhabi [41]. Which then rose to more than 95% at the start of 2013, according to the Abu Dhabi Health Authority, as shown in Figure 2. This rise is attributed to the increase in the level of awareness and knowledge of HPV infection and vaccine among the residents [10,42]. According to the WHO, the available data on female adolescent vaccine uptake for both the first dose and the last dose starts in 2021 and ends in 2024. This explains the gap in data available from 2013 to 2021. In 2021, 46% of female adolescents by age 15 completed the first dose, while 30% completed the last dose. In 2022, 39% of females completed the first dose, while 31% of females completed the last dose. In 2023, 38% completed the first dose and 32% completed the last dose. In 2024, 40% completed the first dose and 37% completed the last dose. Though the percentage of uptake of the first dose by age 15 in females slowly decreased, the percentage of uptake of the last dose increased by six percent from 2021 to 2024. There is no available WHO data to compare the uptake of male adolescent HPV vaccine with that of females aged 15 [43,44].

However, studies have been conducted to assess the acceptance rate and awareness of the HPV vaccine among Emirati men, which could help inform and support the implementation of a national vaccination program for males. The results showed that only 37% of Emirati men had an acceptance rate. This may contribute to the lack of awareness or stigma on the consequences of HPV infection in males, such as HPV causing 39% of penile cancer and 39-65% of head and neck cancers, and up to 88% of anal tumors in both sexes [42]. Lack of awareness and potential stigma against the HPV vaccination in males is a potential barrier that will be further elaborated on.

Comparisons With Global and Regional Utilization

In the GCC, Abu Dhabi (the capital of the UAE) became the first emirate in the Middle East to introduce HPV vaccine coverage in 2008 among all females in both private and public schools. The Abu Dhabi Health Authority of the UAE enforces the use of the quadrivalent HPV vaccine Gardasil [10]. In a cross-sectional study done in the GCC, it was proven that the UAE had the highest vaccination rate at 18.9% in young adults aged 18-39. This shows the awareness and uptake of the HPV vaccine in the nation. Not only is the utilization rate the highest in the UAE, but only the UAE has integrated a successful national program to encourage vaccination against HPV [45].

A systematic review on parental perspectives of the HPV vaccine in the GCC found that the UAE had the lowest HPV positivity rate at 14.7%. The UAE demonstrated a largely favorable attitude toward vaccinating daughters, with 76.6% of parents initially supporting vaccination, a figure that increased to 92.2% when endorsed by official health recommendations. Parents who declined vaccination primarily cited concerns about the vaccine's safety [46]. In 2024, the national immunization program began including HPV vaccines for male adolescents aged 13 to 14 years [47,48]. However, there is no available data on the HPV vaccination status of male adolescents. The FDA integrated the use of a trivalent HPV vaccine for men aged 9-26 in October 2009, reducing the prevalence of genital warts and protecting their spouses against HPV-related infections like cervical cancer [42]. Considering the above FDA approval, it is evident that in the United States, there is HPV vaccine coverage among adolescents of both sexes. Similar to the United States, there has been a substantial decrease in cervical cancer rates due to HPV vaccination coverage [49]. Between 2021 and 2023, the completion rates for the HPV vaccine among 15-year-olds showed slight variations. Among females, 82% received the first dose, and 69% completed the last dose in 2021. In 2022, the rates slightly declined to 80% for the first dose and 68% for the last dose, remaining the same in 2023. For males, 76% completed the first dose and 64% completed the last dose in 2021. In 2022 and 2023, the first dose completion rate increased to 78%, while the last dose completion rate remained at 63% [44]. Compared to the UAE, the United States has a significantly higher HPV vaccination rate among 15-year-old females for both first and last dose completion. A more notable comparison is the vaccination rates among 15-year-old males in the US, as no data is available for this demographic in the UAE [44]. Efforts to improve HPV vaccine uptake are driven by existing public health initiatives [8,50,51].

Factors Influencing the HPV Vaccine Uptake in the United Arab Emirates

HPV vaccination is a vital public health intervention for the purpose of preventing cervical cancer and other HPV-related complications. Nonetheless, the uptake of HPV vaccination among adolescents in the UAE remains suboptimal. Some factors influencing vaccine completion include knowledge gaps, socioeconomic factors, cultural and religious beliefs, and healthcare professionals’ roles.

Awareness: Limited awareness of HPV and its link to cervical cancer remains one of the key barriers to HPV vaccine utilization in the UAE. A study by Ortashi et al. assessing the acceptability of HPV vaccination among male university students in the UAE found that only 31% were aware of HPV [8,23]. Similarly, a study on awareness of HPV infection and vaccination among women in the UAE revealed that only 30% had heard of the virus [10]. Researchers attributed this low awareness to the influence of a conservative society. Furthermore, only 27% of students recognized any connection between HPV infection and cancer [23,52], while just 12% correctly identified HPV as a cause of cervical cancer [46]. This lack of knowledge is largely due to the under-recognition of HPV as a sexually transmitted disease (STD) in developing countries. A cross-sectional study conducted across five MENA regions, including the UAE, further supports this, showing that only 26.9% of participants identified HPV as an STD [22,53]. Awareness of HPV and its vaccine is a crucial factor influencing vaccine utilization. A study conducted across four countries, the UAE, Saudi Arabia, Qatar, and Oman, found that awareness of HPV and its vaccine was generally poor, with UAE participants demonstrating relatively better knowledge, though still at an intermediate level [12,46,54,55]. For instance, a systematic review reported that awareness of the link between HPV and cervical cancer ranged from 11% to 68% in Gulf Cooperation Council (GCC) countries, including the UAE [46,54]. This wide variability underscores the urgent need for targeted educational campaigns to improve knowledge, dispel misconceptions, and enhance HPV vaccine acceptance.

Sociodemographic determinants:* *Sociodemographic factors, including age, marital status, and education, play a crucial role in influencing HPV vaccine uptake in the UAE. Age has been identified as a significant factor in HPV knowledge among women, which may impact vaccine acceptance [23,52]. Younger individuals tend to have greater awareness of HPV and its vaccine, as observed in research conducted among women in Arab communities, including the UAE [12]. Interestingly, rather than a woman’s education being the primary determinant, a husband’s level of education was found to be more strongly associated with better knowledge of HPV infection [10]. Additionally, individuals in the medical field demonstrated higher levels of awareness, suggesting that populations with such backgrounds are more likely to adopt HPV vaccination. While marriage and sexual activity were linked to improved knowledge of HPV infection and vaccination, they did not significantly influence vaccine acceptability among university students [52]. Researchers attributed this to an overall low level of awareness regarding HPV and its vaccine, highlighting the need for broader educational efforts to improve understanding and uptake.

Cultural beliefs:* *Cultural and religious beliefs play a pivotal role in defining a population’s attitudes towards HPV vaccination in the UAE. For instance, when students were asked about the barriers to HPV vaccination, 25% chose objection by a religious authority and 15% chose objection by family members [23,52]. It was also found across the MENA region that parents are hesitant to vaccinate their children for HPV due to the presence of cultural and religious beliefs [16]. Therefore, this stigmatization could lead to low HPV incidence rates and pose a barrier to seeking a means of prevention of complications in the form of vaccination.

Role of healthcare providers: Healthcare professionals play a crucial role in promoting HPV vaccine completion by providing accurate information to parents. Among male university students, 64% identified a doctor’s recommendation as a key factor that would encourage them to receive the vaccine [52]. This highlights the responsibility of healthcare providers to educate patients and guide their medical decisions through informed discussions. Parental hesitancy toward HPV vaccination has also been linked to limited healthcare facilities in the MENA region [53]. This emphasizes the need for public health strategies and interventions that not only improve healthcare access but also encourage parents to vaccinate their children by addressing their concerns and enhancing trust in the healthcare system.

Fear of side effects:* *Concerns about potential side effects remain one of the biggest barriers to HPV vaccination. A study among male university students found that fear of side effects was the primary reason for vaccine hesitancy [23]. This underscores the need for targeted awareness campaigns that not only educate the public on HPV and its risks but also address vaccine safety concerns to build trust and confidence in immunization efforts. Similarly, many parents express anxiety about potential side effects, in addition to fears that vaccinating their children may encourage premarital sexual activity [56,57]. Addressing these misconceptions through clear, evidence-based communication is essential for improving HPV vaccine uptake in the UAE.

Myths and misconceptions:* *Widespread myths and misconceptions about the safety, effectiveness, and necessity of the HPV vaccine pose a significant challenge to its acceptance in the UAE. Common concerns include fears that the vaccine may promote promiscuity or cause infertility. These misconceptions are particularly prevalent in Islamic countries, where cultural and religious beliefs strongly influence attitudes toward sexual health and preventive care [56]. To address this hesitancy, there is a critical need for culturally tailored educational programs that directly tackle these concerns while emphasizing the vaccine’s role in cancer prevention. Clear, evidence-based messaging can help dispel misconceptions and improve HPV vaccine acceptance within the community.

Parental hesitation:* *A study conducted in the UAE found that approximately 14% of parents were hesitant to vaccinate their children against HPV. The primary reasons for this hesitation were concerns about the vaccine's safety and potential side effects [57]. Additionally, cultural beliefs about sexual behavior play a significant role in vaccine hesitancy. Some parents fear that vaccinating their children may imply acceptance of premarital sexual activity. To address these concerns, it is essential to provide clear, evidence-based communication that emphasizes the HPV vaccine’s role in cancer prevention, rather than its association with sexual health. Educating parents on the vaccine’s proven safety and effectiveness can help build trust and encourage higher uptake.

Institutional barriers and healthcare access:* *Beyond cultural and psychological factors, institutional challenges also contribute to the low HPV vaccination rates in the UAE. Key obstacles include vaccine cost, limited availability in certain regions, and inadequate insurance coverage [58]. For many families, the financial burden of the vaccine remains a major barrier, particularly when health insurance does not cover the cost. These structural issues highlight the urgent need for governmental initiatives to improve access, ensure affordability, and expand vaccine availability across the country. Addressing these challenges is essential for increasing HPV vaccine uptake and strengthening public health efforts.

Policy gaps and systemic challenges:* *Systemic challenges further contribute to low HPV vaccination uptake in the UAE. Unlike many other countries with comprehensive national immunization programs, the UAE does not mandate HPV vaccination, which limits coverage rates [59]. Implementing mandatory vaccination policies and integrating the HPV vaccine into the national immunization schedule could significantly enhance vaccine coverage. A more structured and comprehensive approach would ensure that a larger portion of the population, particularly adolescents, benefits from HPV vaccination, ultimately improving public health outcomes.

Social and psychological barriers: Social and psychological factors also play a significant role in vaccine hesitancy. Negative experiences with previous vaccinations can deepen skepticism toward newer vaccines, such as the HPV vaccine [57]. Additionally, misinformation and distrust in healthcare recommendations, particularly when communication is inconsistent or lacks transparency, further discourage vaccine acceptance [60]. To overcome these challenges and increase vaccination uptake, it is essential to build trust between the public and healthcare professionals. This can be achieved through open, honest, and consistent communication, ensuring that individuals receive clear and evidence-based information about the safety, effectiveness, and benefits of HPV vaccination.

Strengthening HPV Vaccination Efforts in the UAE

Expand and strengthen school-based vaccination programs: According to the Advisory Committee on Immunization Practices (ACIP) of the CDC, HPV vaccination is recommended for all males and females aged 11 to 26 years, with the option to administer it as early as age nine and up to age 45 when appropriate [47,48]. Since 2008, the UAE has provided free HPV vaccinations to Grade 11 female students across all schools. Due to the success of this initiative, the program was later expanded to include young women aged 18 to 26. In the same year, the Department of Health (DOH) reinforced cervical cancer prevention efforts by implementing a national screening program [47,48]. However, HPV vaccination for males remains a gap in national immunization efforts, with no dedicated program and limited WHO-reported data on male vaccination rates [43,44]. To achieve broader HPV vaccine coverage in the UAE, efforts should focus on expanding free school-based vaccination to include both genders, strengthening national policies to transition from recommendation to mandatory vaccination for adolescents, and ensuring equal access to the vaccine across all regions. These measures will help improve vaccine uptake and enhance public health protection.

Increase awareness and combat misconceptions: Public awareness plays a critical role in improving HPV vaccine completion. A key challenge is parental hesitancy, often due to concerns about safety, side effects, or misconceptions that HPV vaccination encourages premarital sexual activity [47]. To close this gap, efforts should focus on educating parents about the HPV vaccine’s primary role in cancer prevention, rather than linking it to sexual health. Additionally, clear, evidence-based information should be provided to address safety concerns and highlight the vaccine’s effectiveness and minimal risks. Leveraging mass media campaigns can further enhance awareness by making information accessible, cost-effective, and culturally relevant [8,51].

Ensure equitable access to the vaccine:* *Although the UAE has made progress, barriers such as cost, availability, and insurance coverage continue to limit HPV vaccine accessibility [58]. The financial burden remains a significant concern, especially for families without insurance coverage. To eliminate these barriers, policymakers should expand insurance coverage to include the HPV vaccine, reduce costs through government subsidies or public health funding, and ensure nationwide availability, with a particular focus on underserved regions. These measures will improve accessibility and encourage higher vaccination rates.

Monitor and evaluate vaccination progress: In 2024, the UAE expanded its national immunization program to include adolescents aged 13 to 14 years. However, as of now, no official statistics on this initiative are available on the WHO website [50]. To effectively track progress and enhance HPV vaccination efforts, it is crucial to regularly publish vaccination data to monitor coverage and identify gaps, conduct research and surveys to assess public perception and barriers, and implement targeted interventions informed by data-driven insights. These steps will help refine strategies and improve vaccine uptake.

## Conclusions

HPV is a public health concern with many associated cancers, of which the most notable is cervical cancer. Since 2008, HPV vaccines have proved to be effective; however, adolescent vaccine uptake remains low, especially among males. This review captures numerous factors that affect the coverage of the HPV vaccine in the UAE, such as low knowledge, cultural and religious beliefs, miscommunication on vaccine safety, access to vaccines, and policy constraints. The UAE has made tremendous progress in areas such as school-based adolescent vaccination among females, increased male coverage, enhanced public awareness, and overcoming systemic and social obstacles. Healthcare providers should further encourage vaccine coverage, as well as the implementation of mandatory immunization policies. Increased access to vaccines is key to reaching the WHO global targets for coverage of the HPV vaccine and to lowering the HPV disease burden in the UAE.

## References

[1] Schiffman M, Castle PE (2005). The promise of global cervical-cancer prevention. N Engl J Med.

[2] Ahmed HG, Bensumaidea SH, Alshammari FD (2017). Prevalence of human papillomavirus subtypes 16 and 18 among Yemeni patients with cervical cancer. Asian Pac J Cancer Prev.

[3] Forman D, de Martel C, Lacey CJ (2012). Global burden of human papillomavirus and related diseases. Vaccine.

[4] Gillison ML, Chaturvedi AK, Lowy DR (2008). HPV prophylactic vaccines and the potential prevention of noncervical cancers in both men and women. Cancer.

[5] (2025). World Health Organization: Cervical cancer. https://www.who.int/news-room/fact-sheets/detail/cervical-cancer.

[6] (2025). Centers for Disease Control and Prevention: Cancers caused by HPV. https://www.cdc.gov/hpv/about/cancers-caused-by-hpv.html?CDC_AAref_Val=https://www.cdc.gov/hpv/parents/cancer.html.

[7] Giuliano AR, Nyitray AG, Kreimer AR (2008). Epidemiology of human papillomavirus infection in men, cancers other than cervical and benign conditions. Vaccine.

[8] Elbarazi I, Raheel H, Cummings K, Loney T (2016). A content analysis of Arabic and English Newspapers before, during, and after the human papillomavirus vaccination campaign in the United Arab Emirates. Front Public Health.

[9] Vaccarella S, Bruni L, Seoud M (2013). Burden of human papillomavirus infections and related diseases in the extended Middle East and North Africa region. Vaccine.

[10] Ortashi O, Raheel H, Shalal M, Osman N (2013). Awareness and knowledge about human papillomavirus infection and vaccination among women in UAE. Asian Pac J Cancer Prev.

[11] Zhang J, Ke Y, Chen C (2025). HPV cancer burden by anatomical site, country, and region in 2022. Sci Rep.

[12] Alsous MM, Ali AA, Al-Azzam SI (2021). Knowledge and awareness about human papillomavirus infection and its vaccination among women in Arab communities. Sci Rep.

[13] Odeh HI, Al-Badi SR, Karima B (2023). Exploring the prevalence of Human Papillomavirus (HPV) genotypes in PAP smear samples of women in northern region of United Arab Emirates (UAE): HPV Direct Flow CHIP system-based pilot study. PLoS One.

[14] Krishnan K, Thomas A (2016). Correlation of cervical cytology with high-risk HPV molecular diagnosis, genotypes, and histopathology--a four year study from the UAE. Diagn Cytopathol.

[15] Ali MA, Bedair RN, Abd El Atti RM (2019). Cervical high-risk human papillomavirus infection among women residing in the Gulf Cooperation Council countries: Prevalence, type-specific distribution, and correlation with cervical cytology. Cancer Cytopathol.

[16] Shoja Z, Farahmand M, Hosseini N, Jalilvand S (2019). A meta-analysis on human papillomavirus type distribution among women with cervical neoplasia in the WHO eastern Mediterranean region. Intervirology.

[17] (2025). World Health Organization: Global strategy to accelerate the elimination of cervical cancer as a public health problem. https://www.who.int/publications-detail-redirect/9789240014107.

[18] Garland SM, Hernandez-Avila M, Wheeler CM (2007). Quadrivalent vaccine against human papillomavirus to prevent anogenital diseases. N Engl J Med.

[19] Hall MT, Simms KT, Lew JB (2019). The projected timeframe until cervical cancer elimination in Australia: a modelling study. Lancet Public Health.

[20] Drolet M, Bénard É, Pérez N, Brisson M (2019). Population-level impact and herd effects following the introduction of human papillomavirus vaccination programmes: updated systematic review and meta-analysis. Lancet.

[21] Brisson M, Kim JJ, Canfell K (2020). Impact of HPV vaccination and cervical screening on cervical cancer elimination: a comparative modelling analysis in 78 low-income and lower-middle-income countries. Lancet.

[22] Vincent SC, Al Yaquobi S, Al Hashmi A (2024). A systematic review of knowledge, attitudes, and factors influencing HPV vaccine acceptance among adolescents, parents, teachers, and healthcare professionals in the Middle East and North Africa (MENA) Region. Cureus.

[23] Ortashi O, Raheel H, Khamis J (2013). Acceptability of human papillomavirus vaccination among male university students in the United Arab Emirates. Vaccine.

[24] Luria L, Cardoza-Favarato G (2025). Human Papillomavirus. https://www.ncbi.nlm.nih.gov/books/NBK448132/.

[25] Pennycook KB, McCready TA (2025). Condyloma Acuminata. https://www.ncbi.nlm.nih.gov/books/NBK547667/.

[26] Venkatesan NN, Pine HS, Underbrink MP (2012). Recurrent respiratory papillomatosis. Otolaryngol Clin North Am.

[27] Wichmann G (2017). Variation of HPV subtypes with focus on HPV-infection and cancer in the head and neck region. Recent Results Cancer Res.

[28] Mody MD, Rocco JW, Yom SS (2021). Head and neck cancer. Lancet.

[29] Fowler JR, Maani EV, Dunton CJ (2025). Cervical Cancer. https://www.ncbi.nlm.nih.gov/books/NBK431093/.

[30] Iorga L, Dragos Marcu R, Cristina Diaconu C (2020). Penile carcinoma and HPV infection (review). Exp Ther Med.

[31] Leon ME, Shamekh R, Coppola D (2015). Human papillomavirus-related squamous cell carcinoma of the anal canal with papillary features. World J Gastroenterol.

[32] Del Pino M, Vorsters A, Joura EA (2024). Risk factors for human papillomavirus infection and disease: A targeted literature summary. J Med Virol.

[33] Williamson AL (2023). Recent developments in human papillomavirus (HPV) vaccinology. Viruses.

[34] VIEW-Hub. VIEW-Hub Report (2025). VIEW-Hub: Global vaccine introduction and implementation report. https://view-hub.org/sites/default/files/2025-01/VIEW-hub%20Report_January2025.pdf.

[35] (2025). World Health Organization: Human papillomavirus and cancer. https://www.who.int/news-room/fact-sheets/detail/human-papilloma-virus-and-cancer.

[36] (2025). World Health Organization (WHO): Multisectoral coordination mechanisms operational tool: an operational tool of the tripartite zoonoses guide. May.

[37] Bruni L, Saura-Lázaro A, Montoliu A (2021). HPV vaccination introduction worldwide and WHO and UNICEF estimates of national HPV immunization coverage 2010-2019. Prev Med.

[38] (2025). Gavi: Human papillomavirus vaccine support. https://www.gavi.org/types-support/vaccine-support/human-papillomavirus.

[39] Moreno VA, Nogueira DL, Delgado D (2025). Misconceptions and knowledge gaps about HPV, cervical cancer, and HPV vaccination among central American immigrant parents in the United States. Hum Vaccin Immunother.

[40] Fousseni S, Ngangue P, Barro A (2025). Navigating the road to immunization equity: systematic review of challenges in introducing new vaccines into Sub-Saharan Africa's health systems. Vaccines (Basel).

[41] Al-Nuaimi NS, Al-Ghas YS, Al-Owais AH (2011). Human papillomavirus vaccination uptake and factors related to uptake in a traditional desert city in the United Arab Emirates. Int J STD AIDS.

[42] Al Shdefat S, Al Awar S, Osman N (2022). Health care system view of human papilloma virus (HPV) vaccine acceptability by Emirati men. Comput Math Methods Med.

[43] (2025). HPV Information Centre: Human papillomavirus and related diseases report. https://hpvcentre.net/statistics/reports/ARE.pdf.

[44] (2025). WHO: Human papillomavirus (HPV) vaccination coverage. https://immunizationdata.who.int/global/wiise-detail-page/human-papillomavirus-(hpv)-vaccination-coverage?CODE=ARE+USA&ANTIGEN=15HPVC_F+15HPV1_F+15HPV1_M+15HPVC_M&YEAR=.

[45] Mahmoud I, Al Eid MM, Mohamed MA (2024). Human papillomavirus vaccination and Pap test uptake, awareness, and barriers among young adults in Gulf Cooperation Council countries: a comparative cross-sectional survey. J Infect Public Health.

[46] Alshahrani NZ, Alshahrani JA, Almushari BS (2024). Parental perspectives on human papillomavirus (HPV) vaccination in Gulf cooperation council countries: a systematic review. Medicine (Baltimore).

[47] (2025). WAM: World Health Organization honours UAE for commitment to HPV Vaccine. https://www.wam.ae/en/article/b4oqllw-world-health-organisation-honours-uae-for.

[48] Senior S (2025). Gulf News: MoHAP’s new vaccination policy for school boys: What UAE parents need to know. https://gulfnews.com/uae/mohaps-new-vaccination-policy-for-school-boys-what-uae-parents-need-to-know-1.101890417.

[49] Digital T (2025). Department of Health: Late-stage diagnosis of cervical cancer rate decreases to 14.8%. https://www.doh.gov.ae/en/news/late-stage-diagnosis-of-cervical-cancer.

[50] (2025). Burjeel: UAE cervical cancer cases have seen ‘dramatic drop’ due to proactive measures, says doctor. https://burjeel.com/news/uaes-cervical-cancer-cases-have-seen-dramatic-drop-due-to-proactive-measures-says-doctor/.

[51] (2025). Khaleej Times: UAE: More parents willing to vaccinate their children against cervical cancer, say doctors. https://www.khaleejtimes.com/lifestyle/health/uae-more-parents-willing-to-vaccinate-their-children-against-cervical-cancer-say-doctors.

[52] Wang S, Han B, Wan Y (2020). Do male university students know enough about human papillomavirus (HPV) to make informed decisions about vaccination?. Med Sci Monit.

[53] Garcia MR, Leslie SW, Wray AA (2025). Sexually Transmitted Infections. https://www.ncbi.nlm.nih.gov/books/NBK560808/.

[54] Kurt A, Ekrem EC, Dinç F (2024). Knowledge, attitudes, and beliefs of parents toward the human papilloma virus vaccine. Mediterr Nurs Midwifery.

[55] AlMesbah N, Maatoug J, Selim N, Bougmiza I (2024). Human papillomavirus prevalence and genotypes in Gulf Cooperation Council countries: A scoping review 2017-2024. Qatar Med J.

[56] Kisa S, Kisa A (2024). Religious beliefs and practices toward HPV vaccine acceptance in Islamic countries: A scoping review. PLoS One.

[57] Barqawi HJ, Samara KA, Kannas SM (2024). Vaccine practices, literacy, and hesitancy among parents in the United Arab Emirates. PLoS One.

[58] Tobaiqy M, MacLure K (2024). A systematic review of human papillomavirus vaccination challenges and strategies to enhance uptake. Vaccines (Basel).

[59] Gebreal A, Ashmawy R, Ahmed MJ (2025). A systematic review and meta-analysis on parental uptake and willingness to vaccinate children against human papillomavirus in the Eastern Mediterranean Region. Vaccine.

[60] Ayash C, Raad N, Finik J (2024). Perspectives on human papilloma virus vaccination barriers, knowledge and beliefs, and practices: providers serving Arab-American populations. J Community Health.

